# Cost Saving Analysis of an Enhanced Recovery After Surgery (ERAS) Program for Elective Colorectal Surgery in an ERAS Qualified and Training Center

**DOI:** 10.1002/wjs.12548

**Published:** 2025-03-08

**Authors:** Elisa Bertocchi, Davide Brunelli, Thomas Squaranti, Diego Campagnola, Sara Camparsi, Roberto Tessari, Nicola Menestrina, Irene Gentile, Lorenza Sanfilippo, Nicoletta De Santis, Massimo Guerriero, Giacomo Ruffo

**Affiliations:** ^1^ General Surgery Unit IRCCS Sacro Cuore Don Calabria Hospital Negrar di Valpolicella Italy; ^2^ Hospital Health Direction IRCCS Sacro Cuore Don Calabria Hospital Negrar di Valpolicella Italy; ^3^ Planning and Control IRCCS Sacro Cuore Don Calabria Hospital Negrar di Valpolicella Italy; ^4^ Hospital Pharmacy Unit IRCCS Sacro Cuore Don Calabria Hospital Negrar di Valpolicella Italy; ^5^ Department of Anesthesia, Intensive Care and Pain Therapy IRCCS Sacro Cuore Don Calabria Hospital Negrar di Valpolicella Italy; ^6^ Section of Biostatistics IRCCS Sacro Cuore Don Calabria Hospital Negrar di Valpolicella Italy

**Keywords:** colorectal surgery, cost‐effectiveness, ERAS, preoperative optimization

## Abstract

**Background:**

To ascertain the costs of implementing an enhanced recovery after surgery (ERAS) protocol in elective colorectal surgery throughout all perioperative phases in an Italian ERAS Qualified and Training Center.

**Methods:**

Consecutive patients who had undergone elective colorectal surgery in 2022, the first year of our facility being an ERAS Qualified Center (n 204; ERAS group), were compared to a control group (n 203; pre‐ERAS group) that had undergone elective colorectal surgery in 2017, the last year before the internal ERAS implementation. The primary endpoint was the cost‐effectiveness of the ERAS protocol as determined by evaluating perioperative costs. Secondary endpoints were postoperative clinical outcomes.

**Results:**

In the ERAS group, fewer postoperative complications (*p* < 0.001), a shorter length of stay (LOS) (*p* < 0.001), and a decreased 30‐day readmission rate (*p* 0.047) were reported. The mean cost saving for elective colorectal surgery in the ERAS setting was about €3676.73 per patient. The preoperative costs in the ERAS group were 45% higher than in the control group. The intraoperative phase showed a small but significant decrease in costs (−€324.04, SD 1683.81, and *p* 0.002). The postoperative phase also had a significant decrease in costs (−€3439.30, SD 6903.07, and *p* < 0.001), which was especially apparent in patients with severe complications.

**Conclusions:**

Despite significantly increased costs in the preoperative phase, the ERAS protocol, when highly complied with, may lead to significantly decreased patient pathway costs due to a reduction of postoperative complications, a shorter LOS, and the more targeted use of medication and blood transfusions.

## Graphical abstract

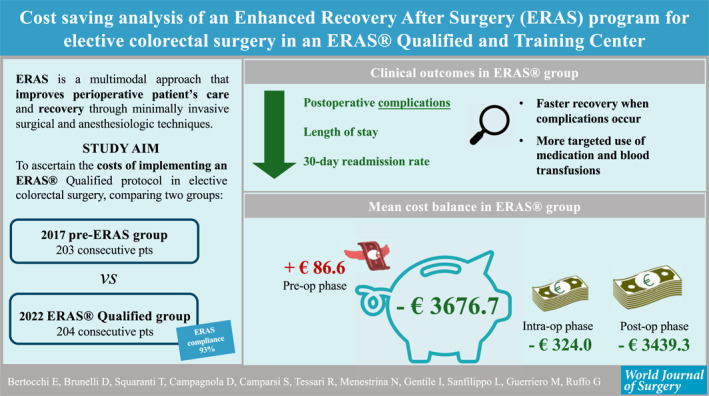

## 1 Introduction

Enhanced recovery after surgery (ERAS) is well recognized as a standardized approach to care for colorectal surgery patients [1, 2, 3, 4]. Several meta‐analyses of randomized control trials have demonstrated a strong reduction in overall postoperative morbidity and in length of stay (LOS) amongst patients following the ERAS protocol [5, 6, 7]. Numerous publications have assessed the cost‐effectiveness of the ERAS protocol in colorectal surgery; however, many are limited to considering LOS costs only [4, 8, 9]. ERAS is a multimodal approach that optimizes the preoperative phase, aiming for minimally invasive surgery, and the enhanced management of anesthesia during the intraoperative phase, culminating in a proactive approach to postoperative recovery [10, 11, 12, 13]. These phases have specific costs relating to staff, the operating room (OR), hospital facilities, devices, medicines, services, and LOS; however, decidedly few studies have considered the costs at each perioperative phase [9, 14, 15]. As such, the aim of this study is to analyze the costs of an ERAS protocol in elective colorectal surgery, during all perioperative phases, in an Italian ERAS Qualified and Training center, with high colorectal surgery volume.

## 2 Materials and Methods

### 2.1 Study Population

The ERAS protocol for elective colorectal surgery was first implemented at IRCCS Sacro Cuore Don Calabria Hospital of Negrar di Valpolicella (VR) in 2018. In 2022, the colorectal unit was certified as an ERAS Qualified Center by the ERAS Society, and in 2023 as an ERAS Training Center.

Consecutive patients who had undergone elective colorectal surgery in 2022 (ERAS group), the first year the colorectal unit was an ERAS Qualified Center, were compared with a control group (pre‐ERAS group) of consecutive patients who had undergone elective colorectal surgery in 2017, the last year before the internal implementation of the ERAS protocol. Data from 2017 were retrieved from the internal institutional register of the general surgery unit in which they were prospectively collected and data from 2022 were downloaded from the ERAS Interactive Audit System (EIAS). This is a retrospective analysis of prospectively collected data. Data of pre‐ERAS and ERAS groups were already collected for other approved studies [3, 13, 16]. IRCCS Sacro Cuore Don Calabria Hospital has a digital program in which it is possible to find all clinical reports of patients who underwent any surgical and medical treatment starting from 2010. Thanks to this system that we were able to create a database of the pre‐ERAS patients, whereas patients of the ERAS group were downloaded using the EIAS dataset.

Inclusion criteria: consecutive patients who had undergone elective colorectal surgery for malignant and benign conditions with or without stoma creation according to a standardized surgical approach in which the surgical devices reported in Table 1 were used; age ≥ 18 years; ASA status I, II, III, and IV; and open and minimally invasive (laparoscopic surgery) surgical techniques. Palliative surgery and urgent/emergency surgery were not considered.

**TABLE 1:** List of care, variables, and connected costs calculated in each perioperative phase.

Pre‐ERAS group *n* 203	Mean cost per patient in euros	ERAS group *n* 204	Mean cost per patient in euros
Preoperative phase			
Standard blood examinations	93.50	Standard blood examinations + anemia and nutritional screening	114.30
ECG	12.55	ECG	12.55
Thoracic scan	25.15	Thoracic scan	25.15
Surgical visit (20 min)	30.39	Surgical visit (20 min)	30.39
Anesthesiology visit (15 min)	30.43	Anesthesiology visit (15 min)	30.43
		Immunonutrition (2 bricks/die for 5 days per patient)	43.16
		Preoperative oral carbohydrate treatment	2.20
		Physiatric visit (20 min)	20.44
Total mean cost per patient in euros (SD)^a^	192.02 (0.00)	Total mean cost per patient in euros (SD)^a^	278.62 (0.00)
Intraoperative phase			
OR—occupation in minutes	459.67	OR—occupation in minutes	397.59
Surgery Laparoscopic Multiuse materials Surgical devices Five single‐use trocars One ultrasonic device One circular stapler One disposable linear stapler One sterilization cycle of a colorectal container Abdominal drain Anesthesia drugs Open Multiuse materials Surgical devices One ultrasonic device One circular stapler One disposable linear stapler One sterilization cycle of a colorectal container Abdominal drain Anesthesia drugs	2180.83	Surgery Laparoscopic Multiuse materials Surgical devices Five single‐use trocars One ultrasonic device One circular stapler One disposable linear stapler One sterilization cycle of a colorectal container Abdominal drain when placed Anesthesia drugs Open Multiuse materials Surgical devices One ultrasonic device One circular stapler One disposable linear stapler One sterilization cycle of a colorectal container Abdominal drain when placed Anesthesia drugs	2164.20
OR occupation in minutes due to eventual postoperative complications	133.69	OR occupation in minutes due to eventual postoperative complications	65.30
Clinical staff Two main surgeons One resident One anesthetist One nurse anesthetist Two surgical nurses	1310.19	Clinical staff Two main surgeons One resident One anesthetist One nurse anesthetist Two surgical nurses	1133.25
Total mean cost per patient in euros (SD)^a^	4084.38 (1471.98)	Total mean cost per patient in euros (SD)^a^	3760.34 (817.61)
Postoperative phase			
Hospital stay in the general surgery unit Costs of housing and fare per day Costs of medical care including surgeons' and nurses' costs Costs of blood and radiological examinations	4173.91	Hospital stay in the general surgery unit Costs of housing and fare per day Costs of medical care including surgeons' and nurses' costs Costs of blood and radiological examinations	1846.52
Hospital stay in intensive care unit (ICU) Costs of housing and fare Costs of medical care costs including anesthetists' and nurses' costs per day	753.10	Hospital stay in intensive care unit (ICU) Costs of housing and fare Costs of medical care costs including anesthetists' and nurses' costs per day	8.33
Blood transfusion costs	145.08	Blood transfusion costs	23.17
Drugs No standard protocol Postoperative opioid IV infusion PRN drugs for pain and nausea Antibiotic prescription by surgeons	309.85	Drugs Multimodal analgesia and nausea‐preventing standard protocol Antimicrobial stewardship (AMS) to optimize anti‐infective therapy	64.63
Total mean cost per patient in euros (SD)^a^	5381.95 (6781.521)	Total mean cost per patient in euros (SD)^a^	1942.65 (1289.70)

Abbreviations: OR, operating room; ECG, electrocardiogram.

^a^ Mean (SD).

The study was conducted according to CHEERS guidelines and a completed checklist was reported.

### 2.2 Objectives

The primary objective of the study was the assessment and the comparison of the cost analysis of an elective colorectal surgery patient's pathway before and after ERAS protocol implementation and certification as an ERAS Qualified Center.

The secondary objectives of the study were to determine and compare the following between the two groups: postoperative complications according to the Clavien–Dindo classification [17]; anastomotic leak (AL); length of stay (LOS); and 30‐day readmission rate and postoperative mortality.

### 2.3 Cost Analysis

Detailed costs for each patient were collected from the hospital accounting database via the healthcare planning and control service using the referral number of every patient included in the study. Costs were divided into preoperative, intraoperative, and postoperative costs and were calculated in euros (€) as shown in detail in Table 1. The costs of the surgical devices were considered as they were billed at the cost price. According to the main objective of the study, that is the evaluation of the impact of an ERAS certified protocol on the costs of the elective colorectal surgical pathway, we adjusted the costs of the facilities and all other costs of 2017 to the costs of 2022 [18]. The costs of the facilities included the crosscutting costs connected to the Hospital such as energy, maintenance, water, heating, and cooling costs.

20% was added to each price to define the costs of the hospital healthcare administration services.

### 2.4 Postoperative Clinical Outcomes Analysis

Postoperative complications were graded according to the Clavien–Dindo (CD) classification [17]. Minor morbidity was defined as a grade ≤ 2 and major morbidity as grade ≥ 3. Postoperative mortality (grade 5) was defined as death during the first 30 days after initial surgery or during the hospital stay [17]. The LOS was calculated from the day of admission to the day of discharge from hospital. The 30‐day readmission rate was calculated by evaluating the number of patients readmitted for a clinical or surgical problem within 30 days of discharge. The compliance rate to the ERAS protocol in the ERAS group was determined from the EIAS as the average compliance to each ERAS item in the perioperative phases [16].

### 2.5 Statistical Analysis

Descriptive statistics, measures of variability, and precision were used to summarize demographic, clinical, and surgical characteristics depending on the type of data (continuous or categorical). Costs were summarized using mean and standard deviation (SD).

Pearson's chi‐squared test, Fisher's exact test, or Wilcoxon rank sum test were performed to compare demographic, clinical, and surgical characteristics, according to the type of data (continuous or categorical), between the two groups of patients (pre‐ERAS and ERAS).

Wilcoxon rank sum test was used to compare costs of the pre‐ERAS and ERAS groups in the three perioperative phases. In addition, Cohen's *d* effect size statistics with 95% confidence interval were reported, together with a qualitative assessment of the magnitude of effect size (with the following thresholds: |d|< 0.2, “negligible”, |d| < 0.5 “small”, |d| < 0.8 “medium”, and otherwise “large”). This analysis was repeated for the following categories of patients: all patients, patients without complications (uneventful postoperative course), patients with complications (any surgical or clinical complications after surgery during the hospital stay and/or after discharge within 30 days), patients with minor complications (CDs 1 and 2), and patients with major complications (CDs 3, 4, and 5).

A *p*‐value of < 0.05 was considered for statistical significance.

Statistical analyses were performed using the software R version 4.3.0. The “effsize” package was used to estimate Cohen's *d* statistics and its interpretation [19].

## 3 Results

### 3.1 Study Population

204 consecutive patients who had undergone elective colorectal surgery, following the ERAS protocol in 2022, were compared to a group of 203 consecutive patients who were treated in 2017 before the implementation of the ERAS protocol. The demographic, clinical, and surgical characteristics of both groups are reported in Table 2.

**TABLE 2:** Patient demographic, clinical, and surgical characteristics.

Characteristic	Pre‐ERAS group *n* 203^a^	ERAS group *n* 204^a^	*p*‐value^b^
Age (years)	64 (18.1)	61 (15.9)	0.018
Sex			0.3
Male	116 (57%)	106 (52%)	
Female	87 (43%)	98 (48%)	
ASA STATUS			0.038^c^
ASA I	50 (24%)	28 (14%)	
ASA II	119 (59%)	144 (71%)	
ASA III	30 (15%)	29 (14%)	
ASA IV	4 (2.0%)	3 (1.5%)	
Type of disease			0.003
Benign	74 (36%)	104 (51%)	
Malignant	129 (64%)	100 (49%)	
Inflammatory bowel disease	27 (13%)	40 (20%)	0.086
Type of surgery			< 0.001^d^
Right colectomy/ileocecal resection	72 (35%)	63 (31%)	
Left colectomy/sigmoidectomy	81 (40%)	82 (40%)	
Hartmann's reversal	0 (0%)	18 (8.8%)	
Total/Subtotal colectomy	6 (3.0%)	9 (4.4%)	
Proctocolectomy	2 (1.0%)	1 (0.5%)	
Proctectomy with ileo‐pouch‐anal‐anastomosis	5 (2.5%)	4 (2.0%)	
Abdominal‐perineal resection	1 (0.5%)	1 (0.5%)	
Anterior resection of the rectum	36 (18%)	26 (13%)	
Surgery duration (minutes)	189.1 (85.2)	178.3 (73.7)	0.2
Laparoscopy	194 (96%)	202 (99%)	0.061
(Missing)	1	0	
Conversion to open surgery	12 (6.1%)	3 (1.5%)	0.015^c^
(Missing)	6	0	
Anastomosis creation	174 (86%)	189 (93%)	0.024
Presence of stoma	60 (30%)	50 (25%)	0.3
Abdominal drainage placement	203 (100%)	117 (57%)	< 0.001

^a^ Mean (SD), *n* (%).

^b^ For continuous variables (“age” and “surgery duration in minutes”), a Wilcoxon rank sum test was performed. For categorical variables, a Pearson's chi‐squared test or Fisher's test were performed according to the characteristics of variables.

^c^ Fisher's exact test.

^d^ Fisher's exact test for count data with simulated *p*‐value (based on 2000 replicates).

### 3.2 Cost Analysis

The cost details in the different perioperative phases are reported in Table 1.

Table 3 shows the cost‐effectiveness analyses between the pre‐ERAS group and the ERAS group in the three perioperative phases comparing patients according to their postoperative outcomes (with or without complications) and evaluating the severity of postoperative morbidity (minor or major) on the cost analysis.

**TABLE 3:** Cost‐effectiveness analyses between pre‐ERAS group and ERAS groups in each perioperative phase comparing the following categories: all patients, patients without complications, all patients with complications, patients with minor complications, and patients with major complications.

Phases	All Patients pre‐ERAS group versus ERAS group	Patients without complications pre‐ERAS group versus ERAS group	All patients with complications pre‐ERAS group versus ERAS group	Patients with minor complications pre‐ERAS group versus ERAS group	Patients with major complications pre‐ERAS group versus ERAS group
Preoperative phase^d^					
Cost difference^a^	+€86.60 (0)	+€86.60 (0)	+€86.60 (0)	+€86.60 (0)	+€86.60 (0)
*p*‐value^b^	—	—	—	—	—
Effect size^c^	—	—	—	—	—
Cohen's *d* coefficient^c^	—	—	—	—	—
Intraoperative phase					
Cost difference^a^	−€324.04 (1683.81)	−€197.92 (1594.98)	−€55.06 (1834.31)	−€253.41 (1148.84)	+€384.15 (1926.67)
*p*‐value^b^	0.002	0.2	0.8	0.2	0.082
Effect size^c^	Small	Negligible	Negligible	Small	Small
Cohen's *d* coefficient^c^	0.27 (0.08, 0.47)	0.19 (−0.05, 0.42)	0.04 (−0.36, 0.44)	0.30 (−0.24, 0.84)	0.26 (−0.87, 0.35)
Postoperative phase					
Cost difference^a^	−€3439.30 (6903.07)	−€2319.89 (6691.28)	−€3739.86 (6473.35)	−€2690.45 (5648.67)	−€4808.20 (7087.81)
*p*‐value^b^	< 0.001	< 0.001	< 0.001	0.034	< 0.001
Effect size^c^	Medium	Medium	Medium	Medium	Large
Cohen's *d* coefficient^c^	0.71 (0.50, 0.90)	0.53 (0.30, 0.77)	0.70 (0.30, 1.11)	0.60 (0.05, 1.13)	0.80 (0.18, 1.43)

^a^ Cost difference: delta of mean costs per patients in euros (SD).

^b^ Wilcoxon rank sum test, comparison between the mean costs in euros of the pre‐ERAS group and the ERAS group.

^c^ Cohen's *d* effect size statistics with 95% confidence interval and qualitative assessment of the magnitude of effect size. The magnitude is assessed using the following thresholds: |d| < 0.2, “negligible”, |d| < 0.5 “small”, |d| < 0.8 “medium”, and otherwise “large.”

^d^ In the preoperative phase, *p*‐value, effect size, and Cohen's *d* coefficient are not reported, since there is no variability in costs in the pre‐ERAS and in the ERAS groups.

**FIGURE 1:**
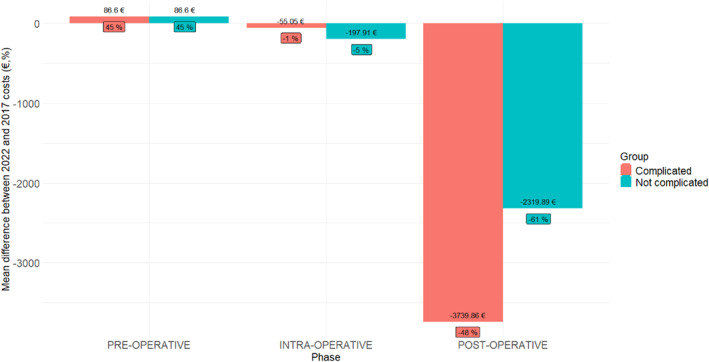
Cost difference of the ERAS group (2022) as compared to the pre‐ERAS group (2017) considering the three perioperative phases in complicated and uncomplicated patients. The value above or below each bar shows how much money the ERAS protocol saved (−) or lost (+) when compared to the pre‐ERAS cohort. Values in euros were calculated as the difference between the costs of 2022 and 2017 (costsof2022–costsof2017), whereas values in percentage represent the relative difference to 2017 costs $\frac{\left(costs\;of\;2022\;–\;costs\;of\;2017\right)}{costs\;of\;2017}\times 100$.

Preoperative costs were higher in the ERAS group with an increase mean cost per patient of €86.60 (Table 3). The preoperative costs in the ERAS group were increased by 45% although the impact of these costs on a patient's pathway was considerable (Figure 1).

The intraoperative phase showed a small but significant decrease in cost per patient (−€324.04, SD 1683.81, and *p* 0.002) (Figure 1). The analysis of the Cohen's *d* coefficient showed an effect size between small and negligible according to the presence or absence of postoperative complications (Table 3).

The postoperative phase demonstrated a significant decrease in cost per patient (−€3439.30, SD 6903.07, and *p* < 0.001) (Figure 1). A medium effect size of the cost saving on uneventful, complicated, and mildly complicated patients was apparent, and a large effect size on patients with severe complications was found (Table 3).

The mean total cost per patient was €9658.34 in the pre‐ERAS group and €5981.61 in the ERAS group (*p* < 0.001), with the mean cost saving for elective colorectal surgery in the ERAS setting being about €3676.73 per patient.

### 3.3 Postoperative Clinical Outcomes Analysis

According to the data collected from EIAS, the perioperative average compliance to the ERAS protocol in the ERAS group was 93%. Specifically, in each phase, ERAS protocol compliance was as follows: 92.9% in the preadmission phase, 100% in the preoperative phase, 79.9% in the intraoperative phase, and 97.9% in the postoperative phase.

Table 4 summarizes postoperative clinical outcomes. Postoperative complications were lower in the ERAS group (*p* < 0.001). Minor and major postoperative complications, according to the Clavien–Dindo classification, were differently distributed between the pre‐ERAS and ERAS groups, with no statistically significant association (*p* 0.5) (Table 4). LOS was significantly reduced in the ERAS group (*p* < 0.001) as was the 30‐day readmission rate (*p* 0.047). There was no significant difference in 30‐day mortality between the two groups (*p* 0.060).

**TABLE 4:** Postoperative clinical outcomes.

Characteristic	Pre‐ERAS group *n* 203^a^	ERAS group *n* 204^a^	*p*‐value^b^
Postoperative complications	81 (40%)	36 (18%)	< 0.001
Severity of complications			0.5^c^
Clavien–Dindo 1	6 (7.4%)	6 (17%)	
Clavien–Dindo 2	36 (44%)	15 (42%)	
Clavien–Dindo 3a	6 (7.4%)	1 (2.8%)	
Clavien–Dindo 3b	27 (33%)^d^	14 (39%)^h^	
Clavien–Dindo 4a	1 (1.2%)^e^	0 (0%)	
Clavien–Dindo 4b	1 (1.2%)^f^	0 (0%)	
Clavien–Dindo 5	4 (4.9%)^g^	0 (0%)	
Anastomotic leak	12 (5.9%)	9 (4.4%)	0.5
Hospital stay (days)	8.0 (7.0)	4.0 (2.0)	< 0.001
30‐day readmission rate	10 (4.9%)	3 (1.5%)	0.047^c^
30‐day mortality	4 (2.0%)	0 (0%)	0.060^c^

^a^ *n* (%), “Hospital stay (days)” expressed as median (IQR).

^b^ For continuous variables (“Hospital stay (days)”), a Wilcoxon rank sum test was performed. For categorical variables, a Pearson's chi‐squared test or Fisher's exact test were performed according to the characteristics of variables.

^c^ Fisher's exact test.

^d^ 9 patients anastomotic leak; 5 patients abdominal collection; 4 patients small bowel perforation; 4 patients bowel obstruction; 2 patients hemoperitoneum; 2 patients *s* urethral injury; and 1 patient evisceration.

^e^ 1 patient anastomotic leak.

^f^ 1 patient small bowel perforation.

^g^ 2 patients anastomotic leak and 2 patients small bowel perforation.

^h^ 9 patients anastomotic leak; 1 patient hemoperitoneum; 2 patients bowel obstruction; 1 patient urethral injury; and 1 patient small bowel perforation.

The potential cost savings of implementing an ERAS protocol in colorectal surgery have been demonstrated in several publications focusing on a reduction of LOS [9]. Implementing an ERAS protocol requires the organization and coordination of hospital personnel around its items and an in‐depth preoperative assessment of a patient's risk factors and treatment pathway; however, these costs are rarely presented in detail in the literature [9, 14, 20]. Such costs are likely the reason for which the ERAS protocol, even if effective in obtaining better postoperative outcomes [21], is not as widespread in its application as it could be [22, 23].

Our study shows a potential cost saving of €3676.73 per patient undergoing elective colorectal surgery following the ERAS protocol.

In the preoperative phase, the hospital spent about 45% more per patient in the ERAS group. These augmented costs were due to the examinations required to ensure high‐quality screening and correction of patient risk factors including malnutrition, anemia, and functional reserves (Table 1). Thorough preoperative optimization was conducted in the ERAS group as demonstrable by high adherence to the preadmission and preoperative items, which were 92.9% and 100%, respectively.

The cost analysis of the intraoperative phase showed a slight but significant decrease in costs for the ERAS group, specifically relating to reduced OR occupation time during initial surgery and any revision surgery due to postoperative complications (Table 3). This is perhaps due to surgeons having more experience in the use of laparoscopy together with an improved organization of patients' pre‐OR preparation. Thanks to the active involvement of the anesthesiologists in the ERAS protocol, all patients of the ERAS group were prepared in the recovery room before entering in the OR where they underwent the peripheral nerve block or eventually epidural/spinal anesthesia and the placement of the arterial access when it was required. This improvement in the perioperative organization allowed an important reduction of the OR—occupation in minutes. Further to this, the reduction in postoperative complications meant revision surgery was required less often. Interestingly, the quality of intraoperative care was higher in the ERAS group due to the use of minimally invasive surgical techniques and optimized anesthesiology management (Table 2). The anesthesiology protocol followed the ERAS items with a focus on goal‐directed fluid therapy, core body temperature monitoring, total intravenous anesthesia, and the prophylaxis of postoperative nausea and vomiting with opioid‐free pain control and nerve blocks, adjuvants, and single‐shot epidural analgesia [24]. Regarding surgical details, as shown in Table 2, in the ERAS group, a higher rate of minimally invasive surgery was associated with a reduced rate of open surgery and lower rates of abdominal drainage placement and stoma creation.

These preoperative investments in the optimization of each patient's status in conjunction with minimally invasive surgery and highly effective anesthesiology management, yielded clinical results in the postoperative phase with a decrease of the following in the ERAS group: postoperative complications (*p* < 0.001), LOS (*p* < 0.001), and 30‐day readmission rate (*p* 0.047) (Table 4). Focusing on postoperative complications, the ERAS group showed lower percentages of CD 3a and no CD 4a‐4b–5, with higher percentages of CD 1 and 3b. The higher percentage of CD 1 was likely related to a better quality of data collection in the ERAS group. Patients undergoing elective colorectal surgery, in adherence with the ERAS protocol throughout the perioperative period, were usually discharged between the second and fourth postoperative days. If discharge was not possible for any reason that did not require any medical, surgical, endoscopic, or radiological treatment, it was recorded in the ERAS group as CD 1. In the pre‐ERAS group, this “non‐linear but uneventful postoperative course” was not considered a complication. The absence of CD 4a‐4b–5 and lower CD 3a percentages was associated with higher CD 3b percentages in the ERAS group that could be explained using the improved approach to all elective colorectal patients who showed a postoperative course with suspected potential for surgical complications. All patients who showed pathological abdominal assessment, typified as having a high DULK score, high C‐reactive protein, and high procalcitonin value [25, 26, 27] in the initial postoperative days underwent an abdominal CT scan and were quickly evaluated for revision surgery. This proactive approach, compared to that used in the pre‐ERAS group, allowed us to quickly identify and address the life‐threatening complications that would otherwise require intensive care. Further to this, a slight increase in CD 3b percentage and a reduction in CD 3a percentage in the pre‐ERAS group correlated with the requirement of radiological drainage of abdominal or pelvic collections.

The better clinical postoperative outcomes that were reported in the ERAS group correlated with a significant cost saving. As shown in Table 3 and in Figure 1, we found a significant reduction of the costs in the postoperative phase in all ERAS patients, especially for patients with major postoperative complications who were considerably reduced in number and had a faster recovery as compared to those of the pre‐ERAS group. The halving of the LOS in the general surgery unit and the clear reduction of intensive care unit occupation were the main reasons for the significant reduction of costs in the postoperative phase. The reduction of postoperative costs was also related to standardized postoperative nausea and pain control protocols that allowed an optimal use of medication as well as the introduction of a successful antimicrobial stewardship program that allowed targeted antibiotic prescription to reduce multidrug resistance and unnecessary utilization of expensive medicine [28]. Further to this, preoperative anemia screening and its correction translated into fewer postoperative blood transfusions, which is good practice especially in colorectal surgery for malignant disease [22, 29].

This study presents some limitations. First, this is a single‐center retrospective observational study and these elements are susceptible to the inherent biases and limitations. Second, our study does not include the costs related to the preoperative diagnostic work‐up and outpatient costs after discharge. Third, the cost details connected to clinical and surgical practices vary by hospitals, and for this reason, financial results found in this study may also vary between different institutions.

## 4 Conclusion

Despite an increase in costs during the preoperative phase and the organizational efforts required for its implementation, the ERAS protocol, in the context of high compliance, can lead to a significant reduction of treatment pathway costs. These cost savings are attributable to a reduction of postoperative complications, a shorter LOS, and the more targeted use of drugs and blood transfusion services. We recommend that the ERAS protocol be used in elective colorectal surgery to not only obtain better postoperative clinical outcomes but to also markedly reduce perioperative costs overall.

## Author Contributions

**Elisa Bertocchi:** conceptualization, data curation, investigation, methodology, validation, writing – original draft. **Davide Brunelli:** data curation, writing – review and editing. **Thomas Squaranti:** data curation, formal analysis, investigation, software. **Diego Campagnola:** data curation, formal analysis, investigation, software. **Sara Camparsi:** data curation, formal analysis, investigation, software. **Roberto Tessari:** data curation. **Nicola Menestrina:** data curation, investigation. **Irene Gentile:** data curation, writing – review and editing. **Lorenza Sanfilippo:** data curation, formal analysis, methodology. **Nicoletta De Santis:** data curation, methodology, software, writing – review and editing. **Massimo Guerriero:** data curation, formal analysis, methodology, software, supervision. **Giacomo Ruffo:** conceptualization, supervision, validation, writing – review and editing.

## Consent

Informed consent was obtained from all individual participants included in the study.

## Conflicts of Interest

The authors declare no conflicts of interest.
